# Assessing Clinician Compliance With DVLA Guidelines for Psychiatric Inpatients

**DOI:** 10.1192/bjo.2024.647

**Published:** 2024-08-01

**Authors:** Samuel Yale, Anastasiia Redko

**Affiliations:** 1 Sheffield Teaching Hospitals NHS Foundation Trust, Sheffield, United Kingdom; 2 The Rotherham NHS Foundation Trust, Rotherham, United Kingdom

## Abstract

**Aims:**

Mental health conditions have wide ranging impacts on individuals, including in their ability to safely drive. Attention, impulse control, judgement, and psychomotor reaction times are some of the ways in which mental health conditions and psychotropic medications impair ability to drive. To ensure safety of patients and other road users, the Driving and Vehicle Licencing Agency (DVLA) provides guidance to clinicians and patients regarding fitness to drive. The General Medical Council (GMC) also states that doctors have a duty to inform patients that their condition and/or medication can impact driving ability.

To evaluate compliance with DVLA and GMC guidelines, an audit was conducted to assess: 1) whether patients’ driving status was established; 2) whether patients were advised to inform the DVLA; and, 3) whether they were advised to inform their insurance company. This was subsequently re-audited after introducing recommendations to improve compliance.

**Methods:**

Each audit cycle reviewed the 30 most recent discharges from an adult general psychiatry inpatient unit before and after intervention. Online notes, multi-disciplinary team (MDT) minutes and discharge summaries were reviewed to assess whether the above criteria were met. Following the initial audit cycle, results were presented at Trust-wide teaching, and driving status was added to an MDT template as a prompt to discuss this with patients. A second cycle was completed four months afterwards.

**Results:**

Results of the first cycle (pre-intervention) showed driving status was established in 73% (n = 22) of patients. Of the drivers, 90% (n = 9) were advised to tell the DVLA, whilst only 9% (n = 1) were advised to tell their insurance company. Post-intervention, 67% (n = 20) of patients had driving status established, whilst 100% (n = 11) of drivers were subsequently advised to inform the DVLA, and 64% (n = 7) advised to tell their insurance company.

**Conclusion:**

Clinicians have a legal and ethical duty to discuss driving status with patients. Failure to do so could have significant consequences on both individual and wider public safety. This audit showed that in clinical practice, key legal requirements were not being fulfilled. Whilst staff education and changes to MDT templates increased the number of drivers being advised to tell the DVLA and insurers, it had little impact on establishing driving status. Therefore, further changes were made to the discharge letter template to remind staff to assess patients’ driving status, and to enable community team follow-up. A third cycle of the audit is currently ongoing to evaluate this change.

